# From stale bread and brewers spent grain to a new food source using edible filamentous fungi

**DOI:** 10.1080/21655979.2020.1768694

**Published:** 2020-05-24

**Authors:** Rebecca Gmoser, Rikard Fristedt, Karin Larsson, Ingrid Undeland, Mohammad J. Taherzadeh, Patrik R. Lennartsson

**Affiliations:** a Swedish Centre for Resource Recovery, University of Borås, Borås, Sweden; b Food and Nutrition Science, Biology and Biological Engineering, Chalmers University of Technology, Gothenburg, Sweden

**Keywords:** *Neurospora intermedia*, stale bread, brewers spent grain, edible filamentous fungi, solid state fermentation

## Abstract

By-products from the food sector with a high load of organic matter present both a waste-handling problem related to expenses and to the environment, yet also an opportunity. This study aims to increase the value of stale bread and brewers spent grain (BSG) by re-introducing these residues to the food production chain by converting them to new protein-enriched products using the edible filamentous fungi *Neurospora intermedia* and *Rhizopusoryzae*. After 6 days of solid state fermentation (at 35°C, with a95% relative humidity and moisture content of 40% in the substrate) on stale bread, a nutrient-rich fungal-fermented product was produced. The total protein content, as analyzed by total amino acids, increased from 16.5% in stale sourdough bread to 21.1% (on dry weight basis) in the final product with an improved relative ratio of essential amino acids. An increase in dietary fiber, minerals (Cu, Fe, Zn) and vitamin E, as well as an addition of vitamin D2 (0.89 µg/g dry weight sample) was obtained compared with untreated stale bread. Furthermore, addition of BSG to the sourdough bread with the aim to improve textural changes after fermentation showed promising outcomes. Cultivation of *N. intermedia* or *R. oryzae* on stale sourdough bread mixed with 6.5% or 11.8% BSG, respectively, resulted in fungal-fermented products with similar textural properties to a commercial soybean burger. Bioconversion of stale bread and BSG by fungal solid state fermentation to produce a nutrient-enriched food product was confirmed to be a successful way to minimize food waste and protein shortage.

## 1. Introduction

Food supply demands resources by means of water, land, nutrients, and energy, and also has been a global environmental concern [1]. With the current projection of the increasing global population and welfare, the food demand will be increased by 70% by 2050 [2], and will have a more severe fingerprint on the environmental impact of the planet [3]. These challenges are exacerbated, considering wasting about one-third of all edible food produced for human consumption [1].

Provision of adequate proteins of animal origin to the growing population generally causes a higher environmental impact than vegetable-based proteins [4]. For example, the livestock sector represents the largest of all anthropogenic land uses, and is responsible for about 18% of all greenhouse gas emissions measured emitted by humans in CO_2_ equivalent. This is a higher share than transportation [5]. Concerns about the ethical and environmental consequences of meat consumption have increased the demand for meat substitutes on the market with products based on legumes and other plants as the leading edge [6]. Unfortunately, not all plant proteins contain all the essential amino acids in the necessary portions. The plants may also contain anti-nutritional compounds that can reduce the protein bioavailability [7]. Protein-rich food attained from filamentous fungal biomass is another alternative on the market. Presently, one company (Quorn®, Marlow Foods, UK) commercializes a product made from the filamentous fungus *Fusarium venenatum* grown in a synthetic medium and mixed with egg albumen, color and flavor compounds to mimic meat [8]. One advantage using filamentous fungi is the texture. These micro fungi produces filaments comparable to meat fibrils with a similar texture to lean meats [9]. To be able to quantitatively measure a food item's texture is very important since texture, along with flavor, nutritional properties, and appearance represents one of the four principal attributes defining food quality [10]. Moreover, the texture of food products is generally regarded as an important factor for acceptability [11].

Filamentous fungal cultivation of plants, cereals, and grains is another promising process to improve the texture, nutritive values, bio-accessibility, and digestibility of plants’ inter alia since the protein profile is altered and antinutritional constituents decrease [12,13]. Two traditional processes on the market using filamentous fungi to convert soybean or peanut press cake into new protein sources are Tempeh and Oncom, originating from the Indonesian cuisine [14]. Both these sources are produced by two commercially exploited filamentous fungi; *Rhizopus oryzae* and *Neurospora intermedia* [13], classified as GRAS (Generally Regarded As Safe), in solid state fermentation (SSF) [15,16]. In Indonesia, Tempehis consumed on a daily basis and, together with other soy products, are one of the top two protein sources in the average diet [17]. These fungi are acknowledged for their deliciousness and high content of protein, with important application as human food [18]. Further advantages include simple nutritional requirements, comparably low levels of nucleic acids, and a taste and smell of the produced fungal biomass which has been reported to be generally pleasant [19]. SSF has re-gained attraction owing its low level of technology, which results in lower capital and operation cost, and the direct application of the fermented product as food with no by-product stream being generated [20]. Another advantage of SSF is the consequence of the different physiology shown by fungi on a solid substrate [21] resulting in higher fermentation productivity and higher end-concentration of products [22]. During the SSF process, enzymes are synthesized and released by the fungi to break down plant cell walls and can thus be applied to improve digestibility of proteins in food by-products while preventing growth of undesirable and pathogenic microorganisms, which is why they have been considered for by-product valorization [23,24]. In this context, SSF has opened a new way of utilizing organic solid by-products and side streams as commercial desirable substrates. Several authors have reviewed applications of SSF by filamentous fungi in the production of different bio-products [25–31], where the local availability, nature, and origin of the solid substrate are important factors affecting the choice of product. Although SSF has been practiced for several centuries in the preparation of traditional food, its application in the production of new food products from food and agricultural by-products is limited.

Within the food industry, the top three major food categories discarded are fruit and vegetables, meat, as well as bakery products. Bread accounts for the major part of the latter group, being a standard part of the diet in the west with a global increase in consumption [26]. Large numbers of bread factories produce a sizable amount of bread waste during processing and distribution [32]. One reason is the short shelf life of bread where changes in textural and sensory attributes, known as staling, occur during storage [33] that is perceived negatively by the consumers even though the bread is still rich in nutrients. In addition, the challenge to always have the shelves at supermarkets filled with newly baked bread throughout the whole day in order to attract consumers contribute to high amount of bread residues every day [26,34]. In European countries, as much as 40% of the annual bread production is estimated to be wasted every year [35]. The increase in bread production puts pressure on waste handling of stale bread, which has become a worldwide concern. Currently, stale bread has no economic value and is commonly disposed of in landfill sites [26], used in anaerobic digestion to recover the energy in the form of biogas, used as a low-value feed component [32] or converted into ethanol [36]. Considering the quality of this bread, there should be more resource-efficient ways to treat this by-product stream. The nutritional value and porosity of wasted bread make it an ideal substrate for fungal SSF processes to produce value-added products. Most studies focus on enzyme production [26,37–41] with little information on the nutritional compositional changes upon fermentation. Yet, in the case the residual bread could still be used for human consumption, SSF by edible filamentous fungi to produce a new food product may be a more sustainable use that allows the whole by-product to be re-used within the food chain [42].

Another underutilized food residue, where its low stability makes the disposal an environmental challenge, is brewers’ spent grain (BSG), the main solid by-product from beer production. BSG, representing the insoluble part of the barley grain after the production of wort, can constitute as much as 85% of a brewery’s total by-product. The relatively high protein and fiber (e.g. β-glucans) content in this food by-product makes it a promising ingredient in food to improve nutritional values and texture [43,44]. Moreover, BSG has been reported as an ideal substrate for SSF by filamentous fungi and has been assessed as a nutrient-enriched feed or in human food formulations [44,45]. Some of the nutritional and functional changes of SSF have been reported; however, studies targeting the important attributes for consumers' choice such as sensory, textural, and organoleptic properties are limited.

Stale bread has previously been shown to be a good substrate for SSF by *N. intermedia* to produce a protein enrich product [46]. However, to the best of our knowledge, the total nutritional composition and textural analysis of a fungal-fermented product from stale bread with and without addition of BSG has not previously been described in the literature. This study extends previous work by examining the nutritional and textural attributes of the fermented product and its suitability as human food compared to other protein sources.

The aim of this study was to show the potential of using stale sourdough bread and BSG as a suitable substrate for SSF by the edible filamentous fungi*R. oryzae* and *N. intermedia*to produce a protein-enriched food product. Stale bread is an excellent underutilized substrate for SSF processes, and the hypothesis was that addition of BSG could add interesting textural changes to the product. Firstly, the nutritional characteristics of stale sourdough bread before and after SSF and on BSG was analyzed. Secondly, the texture of stale bread mixed with 0, 5, 10, 15, or 20 (w/w) % BSG was measured after 6 days cultivation and compared to hamburgers and meat substitutes available on the market. Moreover, the SSF progress was followed over time (0–10 days) by measuring the nitrogen content with and without 10% addition of BSG to the stale sourdough bread to examine how well the substrate mixture was utilized by the fungi. The SSF process holds potential to be implemented at local bakeries, restaurants, or food industries in a small-scale production to recover bread leftovers.

## 2. Materials and methods

### 2.1. Substrates

Steinbrenner & Nyberg (Mölndal, Sweden) kindly provided unsold fresh sourdough bread. The bread was cut into 1–3 cm pieces, air-dried at room temperature for 48 h and milled to ca 2 mm by a laboratory rotor beater mill (SM 100, Retsch Technology GmbH, Germany). After autoclaved, samples were stored in airtight containers in darkness at room temperature prior to use (approximately 2–4 days). Brewers spent grain (BSG) was produced from raw materials from Humlegårdens Ekolager AB, Sollentuna, Sverige, and obtained from Göteborgs Nya Bryggeri AB (Göteborg, Sverige). Freshly collected (wet) samples were washed immediately with distilled water in order to remove any water-soluble sugars, sieved through a pore size of 0.2–0.25 mm, frozen and stored at −18°C. Before use, it was dried at 45°C for 48 h and autoclaved. Composition of the substrates are presented in Table 1.

**Table 1.** Chemical composition of brewers spent grain, and stale sourdough bread before and after 6 days solid state fermentation by *N. intermedia* (based on dry weight). The final composition of the fermented fungus product is also compared with a typical composition of hamburger, commercial mycoprotein product (Quorn®, UK) and soybean burger.

Component		Brewers spent grain [%]	Stale sourdough bread [%]	Fungal product 100% stale sourdough bread	Hamburger minced meat)	Quorn® product	Soybean burger
TAA*		17.2 ± 0.1	16.5 ± 3.4	21.1 ± 0.5			
Total nitrogen		2.7 ± 0.1	2.0 ± 0.1	6.0 ± 0.2	9.1	8.1	3.04
Nitrogen to protein conversion factor		5.5	5.3	5.64			
Lipids		7.7 ± 0.3	2.4 ± 0.2	10.5 ± 1.3	40.4	7.8	23.7
Total carbohydrates^a)^					0	18.5	40.9
Xylans		15.1 ± 0.7	1.7 ± 0.1	1.2 ± 0.1			
Mannans		0.3 ± 0.1	0.4 ± 0.1	3.7 ± 0.1			
Galactans		1.0 ± 0.2	0.5 ± 0.1	1.8 ± 0.1			
Arabinans		6.6 ± 0.3	0.9 ± 0.1	0.8 ± 0.1			
Glucans		27.2 ± 1.8	80.4 ± 0.1	30.7 ± 2.5			
Of which starch		8.7 ± 0.3	65.1 ± 0.2	8.5 ± 0.1	-	-	-
Lignin		45.2 ± 0.1	3.0 ± 0.7	9.6 ± 0.7	-	-	-
Total dietary fiber		58.1 ± 2.1	3.8 ± 1.2	22.0 ± 1.0	0	17.8	8.9
Total solids		98.5 ± 0.1	92.6 ± 0.1	92.5 ± 0.2	-		
Ash		3.3 ± 0.1	3.2 ± 0.1	7.3 ± 0.1	2.9	5.2	7.5
pH		5.7 ± 0.2	4.7 ± 0.2	5.1 ± 0.3	-	-	-
Minerals [µg/g dry weight sample]	Cu	7.22 ± 0.48	4.64 ± 2.43	8.89 ± 0.45	-	-	-
	Zn	46.04 ± 0.35	11.95 ± 0.44	34.12 ± 1.56	120.00	296	20.72
	Fe	30.94 ± 0.03	28.36 ± 0.63	52.17 ± 2.79	43.85	18.50	41.74
Vitamins [µg/g dry weight sample]	D2	0.02 ± 0.00	0.02 ± 0.00	0.89 ± 0.11	0.01	0	0.01
	E^b^	2.82	0.32	27.84	15.64	0	56.18
	C	0.00 ± 0.00	0.00 ± 0.00	0.86 ± 0.45			12.86

^a^Sum of TAA was analyzed on a molar basis minus one water molecule (18 g H_2_O/mol amino acid) that was integrated during the disruption of the peptide bonds [5] in the method used, before converting moles AA into g AA.

^b^Sum of α-, ɣ- and δ-tocopherol.

### 2.2. Fungal strain

*Neurospora intermedia* CBS 131.92 (Centraalbureau voor Schimmelcultures, The Netherlands) and *Rhizopus oryzae* CCUG 28,958 (Culture Collection University of Gothenburg, Sweden), originally isolated from Indonesian food production, Oncom (fermented peanuts) and Tempeh, respectively, were used in the current study. Thus, the strains are edible and therefore Generally Recognized as Safe (GRAS). The fungi were kept on potato dextrose agar (PDA) plates containing: 20 g/L glucose 20, 15 g/L agar, and 4 g/L potato extract. To prepare the PDA plates, inoculated plates were incubated for 4 days at 30°C after which they were sealed with para film and stored in a fridge at 4°C. Inoculum solution of spores was prepared by adding 20 mL of distilled water to each plate followed by releasing the spores from the medium using a disposable plastic spreader. The substrates in all solid state fermentation samples were inoculated with 6.7 (w/w) % *N. intermedia* or *R. oryzae*spore solution with a spore concentration of 2.7 (±0.7) × 10^6^and 1.4 (±0.8) × 10^6^spores/mL respectively.

### 2.3. Fungal fermentation

Solid state fermentation was carried out according to Gmoser et al. [46] with some modifications. Cultivation of both *N. intermedia* and *R. oryzae* were carried out batch wise in sterile petri dishes (100 mm ×20 mm) in which breadcrumbs with and without BSG were added. A total dry weight of 15 g substrate was inoculated with the fungalsporesuspension ofeither*N. intermedia* or *R. oryzae*followed by mixing with a sterile plastic spreader. The initial moisture content was adjusted to 40% (wet basis) with distilled water, and each sample was mixed evenly and then covered with the petri dish lid. Solid state fermentation (SSF) was carried out for 6 days (unless stated otherwise) in a climatic test cabinet (NUVE test cabinet TK 120, Turkey) at90-95% Rh±1% at 35°C under continuous white light (3000 lux). The petri dishes were flipped every second day. Effects of days of fermentation on the nitrogen concentration in the final fungal products were investigated. The addition of 0–20% BSG to the sourdough breadcrumbs used as substratewasinvestigatedinrelation to textural changes of the *R. oryzae* or *N. intermedia* fungal fermented product. Single parameter optimization was used to evaluate nitrogen and texture parameters of the fermented products.

### 2.4. Analyses

The concentration of fungal spore used as inoculum was determined by the help of a Bürker counting chamber. The pH of the initial substrate and final substrate-mycelium mixture was measured by mixing 9 mL of distilled water with 1 g fresh substrate for 4 h at 22°C after which the pH of the suspension was measured [47]. After cultivation, all samples were dried at 45°C until constant weight (10–24 h). Dry weights were determined by further drying at 105°C over night. Before composition analysis, the dried substrates and fermented products were milled to approximately 1.0 mm, using a ball mill (Retsch MM 400, Haan, Germany) in periods of 10 min at a frequency of 30 Hz. Nutritional analyses of hamburger, Quorn burger and soybean burger (Table 1) are based on The Swedish Food Composition Database [48].

#### 2.4.1. Lignin, carbohydrates, and total solids

The total lignin, structural carbohydrates, acetate, and total solids were measured according to the National Renewable Energy Laboratory (NREL) method [49]. Briefly, structural carbohydrates and lignin were applied to a sulfuric acid hydrolysis in two steps. The lignin that was soluble in acid could be quantified spectrophotometrically at 240 nm and the insoluble part was quantified gravimetrically. Starch content was measured following instructions for the total starch assay kit from Megazyme International Ireland Ltd. (Bray Business Park, Bray, County Wicklow, Ireland).

#### 2.4.2. Total dietary fibers

Total dietary fibers were determined as soluble and insoluble fractions according to the enzymatic method AOAC 991.43 combined with Total Dietary Fiber, K-TDFR 06/0 (Megazyme, Ireland).

#### 2.4.3. Total nitrogen and amino-acids

The total nitrogen content in ~0.2 g oven-dried and homogenized material was determined by the Dumas combustion method using a TruMac nitrogen analyzer (LECO, St Joseph, MI, USA) for total nutritional composition represented in Table 1. The total nitrogen content from SSF over time represented in Figure 2 were determined by Kjeldahl digestion using a 2020 Kjeltec Digestor and a 2400 Kjeltec Analyzer unit (FOSS Analytical A/S Hilleröd, Denmark), from ~0.5 g oven-dried and homogenized material. Amino acid analysis was performed according to [50]. The Thermo-Scientific Pierce Amino Acid Standard (20,088) was used for quantification and data present the anhydro-amino acid values [51]. Nitrogen-to-protein (N-to-P) factors were calculated by the ratio of total anhydro-amino acid (TAA) residues to the total nitrogen concentration (TN) of the sample. TN concentration was calculated by summarizing % nitrogen in each AA (N-to-P = TAA/TN).

**Figure 1.**
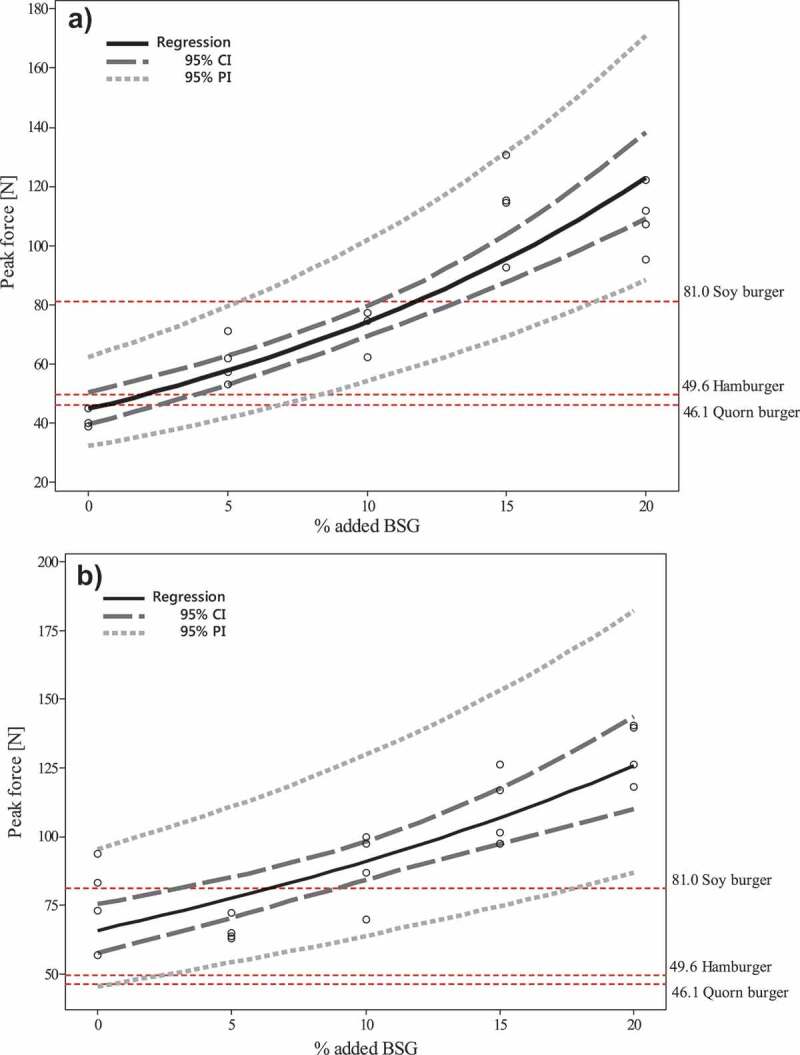
Regression lines from the natural log transformation model obtained of the maximum bite force of (a) fried *R. oryzae* fungal products and (b) fried *N. intermedia* fungal products on stale sourdough bread with addition of 5%, 10%, 15%, or 20% brewers spent grain. SSF was performed for 6 days. A 95% confidence and prediction interval of the estimated regression line and expected interval are shown as dashed lines. The maximum bite force of fried hamburger, Quorn® product, and soy burger are also shown in the figure as dashed horizontal reference lines. The figure also presents the standard error in the estimated average bite force (95% confidence interval) as well as the expected output (bite force value) interval to receive at a specific % of BSG (95% prediction interval).

#### 2.4.4. Fatty acids

Total fatty acid analysis was performed according to Hinchcliffe et al. [50], with the following modifications. Samples were weighed out to around 0.2 g and the method from Lee et al. [52] was applied, comprising chloroform and methanol in a 2:1 ratio. Heptadecanoic acid (C17:0, Larodan AB 10–1700-13) was used for quantification. Total fat was measured gravimetrically following the chloroform/methanol extraction.

#### 2.4.5. Ascorbic acid

Ascorbic acid was extracted from the substrates and fungal product and then analyzed according to Lykkesfeldt [53] using a Decade II Electrochemical detector, ANTEC, with the following modifications. Samples were centrifuged for 4 minutes at 5000 g and then diluted in a mixture of phosphate buffer (50 mM, pH 2.8) and McLvaine buffer (pH 4.5, 0.46 M Na_2_HPO_4_, 0.27 M Citric acid) (ratio 9:1) containing 100 ppm tris(2‐carboxyethyl)phosphine (TCEP). The mobile phase used was 50 mM phosphate buffer at pH 2.8. Ascorbic acid Fluka (95,210) was used for quantitation.

#### 2.4.6. Tocopherol analysis

α, γ, and δ tocopherol was measured according to Larsson et al. [54], with the following modifications. For chromatographic separations, a Phenomenex C18, Luna 100A, 150*3 mm micron column was used. The mobile phase consisted of 98% methanol (HPLC, Laboratory-Scan LtD, Dublin, Ireland) in water, and the flow rate was 0.5 mL/min. Standards used was α-Tocopherol (Sigma Aldrich T3251), γ-Tocopherol (Sigma Aldrich T1782), δ-Tocopherol (Supelco 4–7784).

#### 2.4.7. Vitamin D analysis

Vitamin D was analyzed according to a previously described protocol [55]. 0.3 g of homogenized and lyophilized fungi sample was mixed with 1 g KOH, 5 ml ethanol:methanol (50/50 v/v) with 0.5% (w/v) pyrogallol, and internal standard D6-25(OH)D3 (Sigma Aldrich H-074), blanketed with N_2_ gas, sealed, and shaken at ambient temperature overnight. Then, 5 ml toluene was added, and the sample was treated for an additional 30 min; 2 ml H_2_O was added, and the upper organic phase transferred to a new test tube. The sample was extracted twice with 2 ml petroleum ether:diethyl ether (80:20 v/v). The removed organic phases were pooled, evaporated to a volume of approximately 1 ml, and washed with H_2_O until neutral pH was obtained. The organic phase was evaporated and dissolved in 2 mL 1% 2-propanol in heptane. The extracts were then subjected to solid-phase extraction (TELOS Silica, Kinetics, St Neots, Cambridgeshire, UK) according to the method by Jäpelt et al. [56] and vitamin D2 was analyzed by high performance liquid chromatography-mass spectrometry (HPLC–MS; Agilent 1200 series system with an Agilent 6120 MSD single quadrupole, Agilent Technologies, Santa Clara, CA, USA). The samples were separated on two adjacent C18 columns (Luna, 250 mm, 3 µm C18(2) 100A, Phenomenex, Torrance, CA, USA) by gradient elution with mobile phase A, 98% MeOH and B, Tetrahydrofuran/isopropanol/MeOH (30/30/40%). Gradient started at 100% A for 20 min then 100% B for 15 min, followed by 100% A, total runtime 50 min. Quantification was made against an external standard of Vitamin D2 (Sigma Aldrich 95,220).

#### 2.4.8. Minerals

Samples were mixed with 3 mL water, 150 µL concentrated HCl and 750 µL HNO_3_ in Teflon vials followed by microwave digestion (Milestone microwave laboratory system Ethos Plus Sorisole, Italy) as described previously (Larsson et al., 2007, J. Agric. Food Chem. 2007, 55, 9027–9035 9027). Analysis of minerals was performed using atomic absorption spectroscopy on an Agilent Technologies 200 Series 240FS AA with an UltrAA Boosted Lamp Supply. Quantification was made using standard curves of iron, zinc, and copper standards (Fluka, Buchs, Switzerland).

#### 2.4.9. Ash

Ash content was determined by combustion in a muffle furnace at 550°C for 6 h according to the Approved Method of the American Association of Cereal Chemists, Method 08–01(AACC, 2000).

#### 2.4.10. Texture analysis

Instrumental texture measurement of the fungal products was investigated immediately after 6 days incubation. The raw fungal products were 80 ± 2 mm in diameter and 10 ± 0.5 mm thick. Commercial products were also analyzed for comparison: Quorn vegan burger with 38% (wet weight) mycoprotein (Quorn®, UK), soy burger with 21% (wet weight) soyprotein (Anamma Vegoburger) and minced meat with equal amounts of pork and beef made into hamburger (120 ± 2 g) (ICA, Sweden). All samples were fried at 110°C in 7.5 mL of rapeseed oil for 5 minutes on each side prior to analysis. Shrinkage of the fungal products after frying resulted in a diameter of 65 ± 2 mm and a thickness of 5 ± 1.2.

To simulate a bite force through the products, textural profile analysis (TPA) of the samples was assessed, similarly to Kim et al. [57], butin hexaplicate with a TVT 6700 texture analyzer (Perten Instruments, Sweden with software TexCalc version 4.0.2.50) by cutting the samples. The first bite and its force necessary to penetrate the material have been stated as the keys to assess crispiness, hardness, and tenderness of animal origin food products [58]. A heavy-Duty Stand, knife blade probe with holder and blade set was attached and utilized to measure the force. The samples were perpendicularly oriented to the blade on smooth platform. Sample height was set to 15 mm, and compression 40 mm, i.e. the knife continues 30 mm through the opening in the plate. A pretest, test, and posttest speed of 3 mm/s, 1 mm/s, and 1 mm/s was set, with a trigger force of 50 mN. The force (N) encountered during the ascending movement was recorded 200 times per second until the slab was fractured.

### 2.5. Statistical analyses

Analyses on the raw material (stale sourdough bread and BSG) were taken from one batch (n = 1) and analytical replicates on the fungal products were taken from two batches (n = 2). Three analytical replicates; a = 3 were performed on each batch except for fatty acids that was performed in two analytical replicates; a = 2. For textural analyses, samples were run in five replicates (n = 5) with one analytical replicate; a = 1. Values present the mean value of the measurement ± one standard deviation.

Texture analysis on the *R. oryzae* and *N. intermedia* fungal products were studied with different % of added BSG (full 5 × 2 factorial design) to the stale bread, or fresh versus pre-frozen samples (full 2x2x3 factorial experiment) and with different initial spore concentrations (full 2 × 5 factorial experiment). A mixed factorial regression model with the two fungal species as factor levels and added BSG (or spore concentration or frozen samples) as continuous control was conducted with maximum peak force as output. Raw data from the nutritional experiments and texture measurements were statistically analyzed using the software package MINITAB® (version 17.1.0, Minitab Inc., State College, PA, USA). Since the textural results from the full 5 × 2 factorial experiment with different additions of BSG showed an increased dispersion proportionally to the modeled value, heteroscedasticity, a variance-stabilizing transformation was applied on the response variable. A natural log transformation (Box-cox transformation, λ = 0) of the data (y = ln y) resulted in a transformed model with homoscedastic and well normally distributed residuals. The data obtained were analyzed using a general linear model analysis of variance (ANOVA). A confidence interval of 95% was considered in all analyses. This design made it possible to study different factors simultaneously, as well as their combined effects on the response variable. A regression line, *ln (Peak force (N)) = α×(% BSG)+β* for each fungal species was employed to correlate the response values of the control products with variables attributed to the fungal product.

## 3. Results and discussion

Biotransformation of food industry residues using biotechnology has the potential to reduce the environmental footprint of human activity by the production of an easily accessible protein source and addressing the food waste management issue. Bread is top of the list of our most wasted food items and has a relatively low protein content. In this study, bread industry by-products were converted into a fungal product where mostly carbohydrates were converted into fungal protein and an in-depth nutritional analysis before and after SSF is reported for the first time. The edible filamentous fungus *N. intermedia*successfully improved the nutritional composition of stale sourdough bread after solid state fermentation by an increase in protein and indispensable amino acids, minerals, dietary fiber, vitamin E and enrichment of vitamin D. Nutritional characterization of the substrates and fungal products are presented in Tables 1–3 and discussed further in section 3.1 and 3.2. Since texture is one of the greatest barrier to widespread acceptance for meat alternatives [59], another underutilized industry by-product brewers spent grain was added to the sourdough bread with the aim to improve textural changes after fermentation. Similar textural behavior to a commercial soybean burger, reported for the first time in this study, was attained when stale sourdough bread with 6.5% or 11.8% added BSG was used as substrate to cultivate *N. intermedia* or *R. oryzae* respectively. Figure 1 and section 3.3 presents and discuss the texture analysis further.

**Table 2.** Amino acid profile, total amino acids (TAA), total indispensable amino acids (TIAA), and indispensable amino acid ratio (IAA/AA) in stale sourdough bread based on dry weight referred as ‘before’fermentation begins, after 6 days solid state fermentation (whole fermentation period) by *N. intermedia*referred as ‘after’ and on BSG as a potential substrate. Indispensable amino acids in human are highlighted with gray.

	g AA/100 g sample			AA (% of total AA)			mg AA/g protein^b)^ per day
	Before	After	BSG	Before	After	BSG	Reference pattern**^a)^**
Amino acid profile							
Glycine	0.7 ± 0.0^1^	0.8 ± 0.1^2^	0.7 ± 0.0	3.6 ± 0.1^1^	3.8 ± 0.0^2^	4.1 ± 0.1	
Alanine	0.6 ± 0.0^1^	1.2 ± 0.1^2^	0.8 ± 0.0	3.1 ± 0.0^1^	5.5 ± 0.1^2^	4.5 ± 0.0	
Serine	1.1 ± 0.0^1^	1.2 ± 0.1^2^	0.9 ± 0.0	5.8 ± 0.0^1^	5.6 ± 0.0^2^	5.1 ± 0.1	
Proline	1.8 ± 0.0^1^	1.1 ± 0.1^2^	1.4 ± 0.0	9.5 ± 0.0^1^	5.1 ± 0.2^2^	8.1 ± 0.1	
Valine	1.0 ± 0.0^1^	1.2 ± 0.1^2^	1.2 ± 0.0	5.1 ± 0.0^1^	5.8 ± 0.1^2^	6.8 ± 0.1	39.4
Threonine	0.7 ± 0.0^1^	1.3 ± 0.1^2^	0.9 ± 0.0	3.9 ± 0.0^1^	6.2 ± 0.1^2^	5.1 ± 0.1	22.7
Isoleucine	0.8 ± 0.0^1^	1.1 ± 0.0^2^	0.8 ± 0.0	4.2 ± 0.0^1^	5.1 ± 0.1^2^	4.7 ± 0.0	30.3
Leucine	1.5 ± 0.0^1^	1.7 ± 0.1^2^	1.5 ± 0.0	8.1 ± 0.0^1^	8.0 ± 0.2^1^	8.8 ± 0.1	59.1
Aspartic	0.9 ± 0.0^1^	2.3 ± 0.1^2^	1.4 ± 0.0	4.6 ± 0.0^1^	10.7 ± 0.1^2^	7.9 ± 0.1	
Lysine	0.5 ± 0.0^1^	1.0 ± 0.1^2^	1.0 ± 0.0	2.7 ± 0.0^1^	4.9 ± 0.3^2^	5.6 ± 0.1	45.5
Glutamic acid	6.5 ± 0.1^1^	3.8 ± 0.3^2^	3.8 ± 0.1	35.3 ± 0.1^1^	18.2 ± 0.2^2^	22.0 ± 0.2	
Methionine	0.3 ± 0.0^1^	0.5 ± 0.0^2^	0.4 ± 0.0	1.3 ± 0.0^1^	2.1 ± 0.0^2^	2.0 ± 0.0	22.7 **^c)^**
Histidine	0.5 ± 0.0^1^	0.7 ± 0.0^2^	0.5 ± 0.0	2.4 ± 0.0^1^	3.1 ± 0.1^2^	2.9 ± 0.1	15.2
Phenylalanine	1.2 ± 0.0^1^	1.0 ± 0.0^2^	1.1 ± 0.1	6.5 ± 0.0^1^	4.6 ± 0.0^2^	6.5 ± 0.6	37.9 **^d)^**
Arginine	0.3 ± 0.0^1^	1.4 ± 0.1^2^	0.5 ± 0.0	1.6 ± 0.1^1^	6.7 ± 0.3^2^	3.2 ± 0.2	
Tyrosine	0.5 ± 0.0^1^	0.7 ± 0.0^2^	0.5 ± 0.0	2.4 ± 0.0^1^	3.5 ± 0.1^2^	2.8 ± 0.0	
Cysteine	-	0.3 ± 0.0	-	0.0 ± 0.0^1^	1.2 ± 0.1	0.0 ± 0.0	
TAA	18.4 ± 0.2	21.1 ± 0.5	17.2 ± 0.1	100	100	100	
TIAA	6.3 ± 0.1	8.4 ± 0.2	7.3 ± 0.1	34.2 ± 0.0	39.7 ± 0.0	42.7 ± 0.5	
TIAA/TAA	0.3	0.4	0.3				
IAA score *	0.3	0.4	0.3				

Tryptophan was not analyzed.

* The amino acid score is calculated by a comparison of the content of the limiting essential amino acid in the sample with its content in the requirement pattern based on 2007 FAO/WHO/UNU for adults.

IAA score = (mg of amino acid in 1 g test protein/mg of amino acid in requirement pattern) [60]

^a)^Adult indispensable amino acid requirements based on 2007 FAO/WHO/UNU

^b)^0.66 g protein/kg per day

^c)^Methionin + Cysteine

^d)^Phenylananin + Tyrosine

Same numbers in the same row mean no significant difference between the means at 95% confidence; values are expressed as mean ± standard deviation (n = 2)

**Table 3.** Fatty acids composition of stale sourdough bread based on dry weight before and after 6 days solid state fermentation by *N. intermedia*. Fatty acid (FA) composition, total FA, total saturated fatty acids (SFA), total monounsaturated fatty acids (MUFA), and total polyunsaturated fatty acids (PUFA) in stale sourdough bread based on dry weight referred as ‘before’ fermentation begins, after 6 days solid state fermentation (whole fermentation period) by *N. intermedia* referred as ‘after’ and on BSG as a potential substrate. Essential Fatty acids in human are highlighted with gray.

	mg FA/g sample			FA (% of total FA)		
	Before	After	BSG	Before	After	BSG
Fatty acid profile						
C 12:0	0.00 ± 0.00^1^	0.06 ± 0.03^2^	0.00 ± 0.00	0.01 ± 0.01^1^	0.19 ± 0.08^2^	0.00 ± 0.00
C 13:1	0.03 ± 0.00^1^	0.00 ± 0.00^2^	0.00 ± 0.00	0.12 ± 0.00^1^	0.01 ± 0.00^2^	0.00 ± 0.00
C 14:0	0.05 ± 0.00^1^	0.28 ± 0.11^2^	0.00 ± 0.00	0.22 ± 0.00^1^	0.84 ± 0.23^2^	0.01 ± 0.00
C 15:0	0.06 ± 0.00^1^	0.13 ± 0.01^2^	0.00 ± 0.00	0.26 ± 0.00^1^	0.42 ± 0.02^2^	0.01 ± 0.00
C 16:0	5.15 ± 0.25^1^	5.01 ± 0.75^1^	1.40 ± 0.04	23.35 ± 0.02^1^	15.75 ± 0.28^2^	19.83 ± 0.05
C 16:1n7	0.03 ± 0.01^1^	0.22 ± 0.05^2^	0.01 ± 0.00	0.14 ± 0.02^1^	0.69 ± 0.06^2^	0.14 ± 0.02
C 17:1n7	0.01 ± 0.00^1^	0.08 ± 0.01^2^	0.00 ± 0.00	0.06 ± 0.01^1^	0.27 ± 0.02^2^	0.02 ± 0.00
C 18:0	0.47 ± 0.02^1^	1.34 ± 0.16^2^	0.09 ± 0.00	2.13 ± 0.01^1^	4.23 ± 0.05^2^	1.25 ± 0.01
C 18:1 n9/n12	2.83 ± 0.15^1^	7.81 ± 1.23^2^	1.27 ± 0.02	12.82 ± 0.04^1^	24.51 ± 0.62^2^	17.98 ± 0.19
C 18:1 n7	0.11 ± 0.01^1^	0.21 ± 0.06^2^	0.06 ± 0.00	0.49 ± 0.02^1^	0.66 ± 0.09^2^	0.81 ± 0.02
C 18:2 n6	13.07 ± 0.63^1^	13.73 ± 1.62^2^	4.15 ± 0.10	59.23 ± 0.15^1^	43.26 ± 0.62^2^	58.77 ± 0.03
C 18:3 n3	0.01 ± 0.01^1^	1.74 ± 0.11^2^	0.00 ± 0.00	0.04 ± 0.03^1^	5.63 ± 0.41^2^	0.01 ± 0.01
C 20:0	0.00 ± 0.00^1^	0.13 ± 0.02^2^	0.00 ± 0.00	0.01 ± 0.00^1^	0.42 ± 0.02^2^	0.01 ± 0.01
C 20:1 n9	0.02 ± 0.00^1^	0.08 ± 0.02^1^	0.00 ± 0.00	0.09 ± 0.01^1^	0.26 ± 0.03^2^	0.04 ± 0.00
C 20:2 n6	0.00 ± 0.00^1^	0.05 ± 0.00^2^	0.00 ± 0.00	0.01 ± 0.01^1^	0.17 ± 0.00^2^	0.04 ± 0.01
C 20:3 n3	0.03 ± 0.01^1^	0.30 ± 0.05^2^	0.00 ± 0.00	0.06 ± 0.01^1^	0.98 ± 0.28^2^	0.01 ± 0.01
C 20:5 n3	0.00 ± 0.00^1^	0.01 ± 0.00^2^	0.00 ± 0.00	0.01 ± 0.01^1^	0.02 ± 0.00^2^	0.01 ± 0.00
C 22:0	0.06 ± 0.01^1^	0.16 ± 0.01^2^	0.01 ± 0.01	0.29 ± 0.01^1^	0.5 ± 0.05^2^	0.11 ± 0.11
C 22:5 n3	0.00 ± 0.00^1^	0.01 ± 0.00^2^	0.00 ± 0.00	0.00 ± 0.00^1^	0.04 ± 0.02^1^	0.01 ± 0.01
C 24:0	0.00 ± 0.00^1^	0.19 ± 0.01^2^	0.00 ± 0.00	0.02 ± 0.02^1^	0.59 ± 0.04^2^	0.01 ± 0.01
C 24:1 n9	0.00 ± 0.00^1^	0.05 ± 0.01^2^	0.00 ± 0.00	0.00 ± 0.01^1^	0.16 ± 0.01^2^	0.01 ± 0.00
Total FA	22.06 ± 1.11	31.78 ± 4.21	7.07 ± 0.17	100	100	100
Total SFA	5.80 ± 0.30	7.37 ± 1.12	1.50 ± 0.05	26.29 ± 0.02	23.65 ± 0.46	21.27 ± 0.12
Total MUFA	3.13 ± 0.18	8.53 ± 1.39	1.39 ± 0.02	14.19 ± 0.09	27.36 ± 0.83	19.65 ± 0.26
Total PUFA	13.13 ± 0.64	15.88 ± 1.71	4.18 ± 0.11	59.52 ± 0.10	50.27 ± 1.29	59.08 ± 0.14

Same number in the same row mean no significant difference between the means at 95% confidence; values are expressed as means±SD (*n* = 2).

The simple fermentation process together with cheap and abundant substrates used in this study provides opportunities for homes, restaurants, and industries to reduce their waste streams while producing new food products based on filamentous fungi.

### 3.1. Characterization of stale sourdough bread and brewers spent grain

Stale bread is an interesting candidate as a substrate for solid state fermentation (SSF) being porous in its structure with easily accessible nutrient for the fungi to consume. The sourdough bread used in this study is mainly composed of starch, 65.1%, and has a total amino acid (TAA) content of 16.5%, (Table 1). The quality of the protein was estimated by analyzing the amino acid profile. The presence of indispensable (essential) amino acids (IAA) in food is important in the human diet, since IAA cannot, or are poorly able to be synthesized in human tissues [60]. Table 2 shows the amino acid profile of stale sourdough bread.

Brewers spent grain (BSG) resemble a more recalcitrant structure compared to stale bread. However, with a TAA content of the BSG of 17% and a high dietary fiber content of 58.1, it is a promising supplement or substrate from a nutritional and textural perspective [61] that can add value to a new food product, Table 1. The amino acid profile of BSG revealed a total IAA out of total AA of 43% (Table 2) indicating beneficial addition of amino acids if combined as substrate for SSF. Fungal SSF for BSG valorization have previously been investigated as a source of protein for feed or in food formulations.

### 3.2. Fungal fermentation on stale bread

The nutritional composition of stale sourdough bread successfully improved after 6-days SSF by *N. intermedia*. The starch in sourdough bread was replaced as the dominant element by protein (21.1 ± 0.1% total amino acid residues) after fermentation (Table 1). The results are in agreement with similar studies aiming to improve the nutritional value of wheat bran, sugar beet pulp, citrus waste [62], and red quinoa seeds [63] through SSF by *Neurospora* sp. The protein content of the solid substrates increased from 13%, 15%, 7%, and 13% to 30%, 30%, 18.2%, and 21% on a dry weight basis, respectively [62,63]. An increase in protein content after SSF is a result of the fungal metabolic activity during growth and substrate decomposing into H_2_O andCO_2_ [15,64]. The fermented fungal product was also compared with Quorn vegan burger from mycoprotein (Quorn®, UK), soyprotein burger (Anamma Vegoburger) and minced meat with equal amounts of pork and beef made into hamburger, all available on the market. The final protein content after SSF is higher than the value reported in the commercial soybean burger (19% protein) but did not reach the same protein level as hamburger and Quorn burger (57.0% and 50.7% crude protein, respectively).

#### 3.2.1. Amino acid profile

The sum of total indispensable amino acids (TIAA)/g of total amino acids (TAA) also increased after SSF (Table 2).The amino acid profile after SSFis in agreement with those reported by [65] for pure *N. intermedia* biomass. The decrease in selected AA after SSF, mainly glutamine, proline, and the indispensable AAphenylalanine, may be attributable to the preferential utilization of these AA by *N. intermedia*. In both cases, methionine was the limiting AA. However, a slight increase was observed after SSF. Similar observations were reported by Ibarruri et al. using BSG as the sole substrate in SSF with *Rhizopus* sp. After 9 days SSF, the overall EAA content in fermented BSG was 1.5 times higher than in the unfermented BSG with a decrease in specific AAs content such as glutamine, proline, phenylalanine, and leucine while methionine increased. Moreover, the total amino acid content after 9-days SSF on tapioca by-product and tofu waste by *N. crassa* [66] as well as on durian and tofu waste by *Phanerochaete chrysosporium* and *N. crasssa* [67] was higher than before fermentation with an increase in methionine. Methionineis important nutritionally since the concentration has been reported to be marginal in legume proteins and is below the recommended value in the stale sourdough bread. Methionine, as well as other sulfur containing AAs has also been related to support the characteristic umami flavor [68]. One interesting study demonstrated that *N. crassa*-lactobacillus interaction in solid state co-fermentation enhanced the utilization of agro-food wastes and enriched the fermented product with protein and essential amino acids compared to using *N. crassa* alone [69]. The relatively high content of lysine (4.9 ± 0.3 g/100 g sample) obtained in the fungal product improved the AA profile since lysine is stated as one of the limiting amino acids in plant-derived proteins [57], especially from wheat [60]. The increase in threonine after SSF is also of particular nutritional importance, since it is present in low concentrations in cereal proteins. To the best of our knowledge, this is the first work describing the amino acid profile change before and after SSF of stale sourdough bread by edible filamentous fungi, which contributing to important information for use as a food product. However, it is important to comment that nutritional value primarily defines both AA composition and digestibility [60]. Filamentous fungal biomass has previously been stated to contain appreciable quantities of IAA required for human nutrition as well as a favorable protein-digestibility-corrected AA score compared to beef [70].

#### 3.2.2. Fatty acids profile

The total lipid content increased after cultivation, from 2.4% in sourdough bread to 10.5% in the fungal-fermented product, Table 1, with a shift to more monounsaturated fatty acids, Table 3. The most abundant fatty acid in the fermented product was C18:2 followed by C18:1, which almost doubled after SSF compared to the stale sourdough bread. The final lipid content and shift in individual fatty acids is in agreement with previously reported values from pure *N. intermedia* biomass cultivated in thin stillage as substrate [71] and solid state fungal cultivation on sweet potato [72], respectively. However, other authors have reported a decreasein total lipids after *Rhizopus* sp. SSF on rice bran and *N. intermedia* SSF on okara due to fungal lipase activity for biomass production [73–75]. The increase in the total lipid content may be due to the fact that a high concentration of carbohydrate in stale bread favors the production of mycelial fat [76] or, as stated by Beuchat and Worthington [77], due to the selective utilization of non-lipid materials during cultivation. The fungus *N. sitophila*, among other fungi, was reported by the same authors to not utilize lipids during SSF of full-fat peanuts and preferential utilization of free fatty acids was not detected during the cultivation phase. Meanwhile, C16:0, C18:2 n6 and C13:1 decreased after 6 days cultivation, indicating preferential utilization of those fatty acids and production of lipids by the fungus during cultivation. The decrease in C16:0 and increase in C18:3n3 could be explained by the fatty acid biosynthesis pathway in *Neurospora*. The biosynthesis begins with the formation of C16:0, which is elongated to C18:0 and is then desaturated to form C18:1n9, C18:2n6 and subsequently C18:3n3 [78]. However, it is unclear whether C18:1 is from de novo lipogenesis or partly from saturation of C18:2, sincea proportional reduction of C18:2 was accompanied with increase in C18:1 [72].

The essential fatty acid α-linolenic acid (ALA; C18:3n3) has been suggested to play a role in cardiovascular disease reduction [79], which supports the importance of ensuring adequate intake through the diet. An increase of ALA from 0.04% to 5.6% of total fatty acids was seen after SSF. The fatty acid profile, reported for the first time on fermented stale bread by *N. intermedia*,has potential to be altered further by changing processing parameters [70] to promote, e.g. ALA production.Čertík et al. [80] investigated the ability of four *Mucor* strains to enrich various cereal-based products with γ-linolenic acid (GLA). Results revealed that supplementing the substrate with glucose or by addition of spent malt grain to the substrates in a ratio of 3:1 significantly increased fungal GLA production since the added spent malt grain improved the porosity of the substrates, which resulted in better respiration and aeration efficiency. In this study, addition of BSG may possess similar responses.

#### 3.2.3. Micronutrients

There was a significant increase in mineral content including iron, zinc, and copper after SSF on stale sourdough bread by *N. intermedia*but the ratio between the minerals stayed relatively similar (cf. Table 1). Similar phenomenon was observed by Oliveira et al. [81] in relation to *R. oryzae* using rice bran as substrate, they found an increase of 30% in total ash content after 48 h of fermentation. As for nitrogen, the increase of minerals is a result of a considerable reduction in total dry weight after fermentation. Since the absorption of iron and zinc are negatively influenced by phytic acid (found in the outer layer of cereal grains) and positively influenced by animal protein, the mineral level in meat substitutes is of great importance for people following a vegan or vegetarian diet [82]. Considering the mineral levels of BSG, especially for zinc (46.0 ± 0.4 µg/g dry weight) an addition to the sourdough bread has potential to increase the nutritional value.

A major increase in the fat-soluble antioxidant, vitamin E (α-, δ- and ɣ-tocopherol) was obtained after fermentation resulting in 27.8 ± 0.3 µg/g dry sample with a more or less equal contribution from α-, δ- and ɣ-tocopherol, Table 1. To the best of our knowledge, this is the first time the production of vitamin E has been reported to be produced by *N. intermedia*. The vitamin E content is higher than reference products;15.6 and 0 µg/g dry hamburger and Quorn®, respectively. According to European Food Safety [83], an adequate intake for the fat-soluble antioxidant, vitamin E (α-, δ- and ɣ-tocopherol) is set at 13 and 11 mg/day for men and women, respectively. The most important source of vitamin E in human diets are vegetable oils where the dominant dietary form is ɣ-tocopherol. However, the most biopotent form of vitamin E is α-tocopherol [84].

Vitamin D, which is the generic term for ergocalciferol (vitamin D2) and cholecalciferol (vitamin D3), was also analyzed in the fungal product. Ergocalciferol, produced by the fungus from its provitamin ergosterol in the presence of ultraviolet-B (UV-B) irradiation, was detected after solid-state fermentation. As much as 0.89 ± 0.1 µg/g dry weight was obtained in the final product, after SSF which is significantly higher than the hamburger, soybean burger, and Quorn burger containing 0.01, 0.02, and 0 µg/g, respectively (Table 1). A patent for improving the vitamin D2content of fungi claimed that exposure to pulsed UV-B irradiation at 16 seconds increased the vitamin D2content in dried oyster mushroom powder by 185% compared to no UV-B treatment resulted in 24 µg/g dry weight. In the European Union, the Adequate Intake (AI) for vitamin D is given as 15 µg/day [83]. Fungi are the only vegetarian/vegan natural food source of vitamin D and most food contain very low amount of vitamin D. Since an estimated amount of 40–90% of adults worldwide have vitamin D deficiency [84], new food products naturally high in this vitamin is highly interesting.

#### 3.2.4. Fiber content in the fungal product

Dietary fibers are recommended to be part of a healthy diet, yet intake continues to be less than recommended levels for many people around the world [85]. For an average adult, 25–35 g of fibers per day is recommended by The Swedish Food Agency [86]. *N. intermedia*SSF on the stale sourdough bread exhibited a noticeable high value of total dietary fiber after 6 days, constituting as much as 22 ± 1% on a dry weight basis, Table 1. As regards the recommended daily fiber intake, it is met by consuming 114–159 g of the fungal-fermented product. Similarly, a study by Canedo et al. [45] reported a final crude fiber content of 22% on a dry weight basis after 7 days of solid state fermentation of BSG by *R. oligosporus*.Likewise, SSF of red quinoa seeds with *N. intermedia* resulted in an increase in total fiber by 73% in the final product. The increase in dietary fiber after fermentation is mainly attributed to components of the fungal biomass. Fungal cell walls components, such as β-glucans, chitin, and chitosan have recently been classified as parts of beneficial dietary fiber in foods [63]. Fibers have also been reported to improve sensory properties such as texture in meat analogs [87].

### 3.3. Texture analysis

By only using the sourdough bread as substrate, a nutritious enriched fungal product was attained, however, another important attribute for consumers' acceptance is texture. Yet, studies on textural measurements of SSF products from fungi for food applications are scarce in the literature. The BSG analyzed in this study contained 58 ± 2% dietary fiber (dry weight basis), and was therefore investigated as an additional substrate to the sourdough bread to introduce textural changes in the final product that can resemble commercial protein alternatives on the market. In this study, the fungal product with about 0–12% added BSG using any of the two filamentous fungi showed acceptable textural properties. As expected, the maximum force increased with increasing BSG concentration in the substrate. *N. intermedia*product with 0–20% addition of BSG resulted in a higher peak force compared to *R. oryzae*, ranging between 66–130 N and 42–109 N, respectively (Figure 1). There is a significant effect on bite force from % BSG and the fungal species used as well as interaction between the substrate and fungal biomass. Regression equations of ln(Peak force (N)) = 4.1852×(%BSG)+0.032 and ln(Peak force (N)) = 3.8004 ×(%BSG)+ 0.051 was obtained for *N. intermedia* and *R. oryzae* respectively. This also implies an exponential increase of the bite force as a function of BSG content. The commercial control products resulted in a bite force of 50, 46, or 81 N for hamburger, Quorn® and soybean burger. Based on the regression, the most similar textural behavior to the soy burger was shown to be fungal products made from stale sourdough bread with 6.5% BSG cultivated by *N. intermedia*, or stale sourdough bread with 11.8% added BSG cultivated by *R. oryzae*. Furthermore, *R. oryzae* with 2.1% added BSG was shown to simulate the texture of a hamburger (Figure 1). The filamentous mycelium shape together with the BSG recalcitrant structure potentially simulates meat fibers, which contributes to the texture of the fungal product. A study by Waters et al. [61] reported that replacing up to 10% of the flour in bread with BSG or BSG fermented with *L. plantarum* improved the nutritional properties of the bread while still accepted sensory and textural attributes. Addition of minor ingredients to further improve the final texture to meet the consumer’s expectations of the fungal product is possible.

The effect of freezing the fungal products for 24 h before frying the samples as above on final maximum peak force was also studied. A significant difference between fresh or frozen samples was only observed for *R. oryzae* fungal products. By freezing before frying, the maximum bite peak force increased by an average of 15 ± 6 N for all *R. oryzae* fungal product samples with the highest increase for fungal products with only sourdough wheat bread used as substrate. According to the British company Rank Hovis McDougall (RHM) (commercialized by Quorn®), freezing the fungal product potentially result in a more similar texture to meat because the ice crystals formed push the fibers together, creating bundles [88].

The objective textural data contribute to important knowledge about the products quality attributes. Bite force test is suggested to be helpful indices for estimating the chewing properties of a food product; however, it is still not clear if a human’s sensory perception of hardness can mainly be based on the applied force. However, supplementation with up to 10% BSG in bread has previously been reported to be at a sensorial acceptable level (Waters et al., 2012).

### 3.4. Nitrogen content over times with addition of BSG

**Figure 2.**
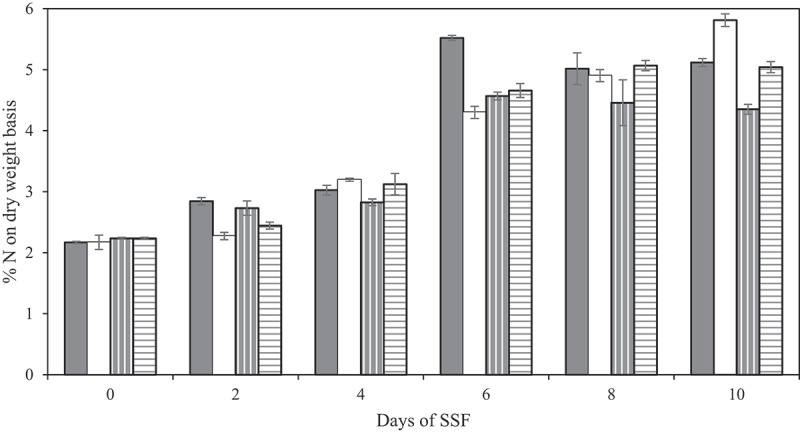
Percent nitrogen (n) based on dry weight of the fungal product after SSF on stale sourdough bread (filled column) or stale sourdough bread and 10% brewers spent grain (pattern columns) using *N. intermedia* (gray background) or *R. oryzae* (white background) after 0–10 days fermentation under light at 35°C, 90% Rh, and 40% initial moisture content. Results are expressed as the mean value ± one standard deviation.

The protein increase is one of the most interesting effects of SSF that convert the substrate into a high value alternative protein source. Since up to 10% addition of BSG to the stale sourdough bread was shown to improve the texture in the fungal product, it is important to study how the protein content changes over time with the new substrate mixture. Therefore, nitrogen content during SSF with *N. intermedia* and *R. oryzae* of stale sourdough bread with or without addition of 10% BSG was followed over 10 days. Already after 5 days SSF, fungal mycelia completely covered the substrate of all samples and a mat of white or yellow/orange mycelium appeared on the surface. Similar observations were also carried out by Melikoglu et al. [37]. The fungal products smelled similar to fresh mushrooms and with a pleasant nutty and fruity smell and none of them showed any sign of contamination. Different strains of *Neurospora*haveprevious been stated to be related to fruity and almond-like aromas [89,90]. However, SSF for more than 6 days resulted in less pleasant smell and appearance. Furthermore, after 6 days SSF and increasingly over longer fermentation time, fungal sporulation was observed at the edges of the petri dish where aerial hyphae are formed in direct contact with the air. Generally, adequate aeration is one of the factors necessary for spore formation. The stale bread packed in petri dishes with lid probably created a less aerobic condition unfavorable for sporulation, while the increased moisture level during fermentation could also limit fungal sporulation [91,92]. After the fermentation was terminated, the fungi products remained visually appealing up to 24 hours at room temperature, after which the product softened by a reduced sponginess (visual inspection) and fungal sporulation appeared on the surface. However, the shelf life has to be examined further, comprising, e.g. microbial and chemical analyses, to be properly determined. As stated in section 3.3, freezing the final product and re-thawing it did not influence the texture of the final product for *N. intermedia* but increased the bite force for *R. oryzae* after frying. Since the process did not change the appearance, freezing is suggested as the best option for storage to extend the shelf life. Substrates with only sourdough bread resulted in the highest final N content, Figure 2. Nitrogen content increased during fermentation for both fungi up to 6 days. For *N. intermedia* it reached its maximum nitrogen value of 5.5 ± 0.1% at this point (corresponding to 31.1 ± 0.2% protein (N-to-P conversion factor 5.64)) then decreased. The same trend followed for 10% BSG. Results are in agreement with Melikoglu et al. [93] stating that 6-days fungal SSF on bread crumbs was shown to be optimal for enzyme production. After fungi growth declines, sporulation, and ammonia production due to protein breakdown appears [94]. The nitrogen content of *R. oryzae* product on stale sourdough bread continued to increase and 5.8 ± 0.1% nitrogen was obtained after 10 days SSF corresponding to 32.8 ± 0.6% protein, whereas samples with 10% BSG flattened out after 8 days SSF. Another study focusing on cultivating of *R. oryzae*on rye flakes and soya grits resulted in a final protein content of 36% after 4 days SSF [15]. Considering the above, it is believed that the best time for terminating the cultivation is after six or 8 days SSF for *N. intermedia* and *R. oryzae*, respectively, especially as the increase in protein between eight and 10 days was marginal.

SSF by *N. intermedia* or *R. oryzae*on BSG as the sole substrate was also investigated in this study (data not shown). The final protein content of the product with only BSG corresponds to 21.4% and 24.2% (N-to-P conversion factor 5.49) respectively, resulting in products with orange mycelium and spores or a white mycelium mat covering the BSG particles, which indicate that both fungi were able to utilize BSG as substrate. Similarly, Ibarruri et al. reported a final protein concentration of 31% after 8 days solid state fermentation of BSG using *Rhizopus* sp. containing up to 31.7% protein with an improved amino acid profile. Since both *N. intermedia* and *Rhizopus* sp. are able to degrade phytate present in BSG [45,63], the problem of using BSG as food due to its high level of phytic acid [43] can be overcome through SSF. Subsequently, even though addition of BSG did not increase the protein content over the 10 days period, it is suggested to be incorporated into fermentation products in ratios below 10% to develop the texture and to process BSG by fungal fermentation to improve its digestibility and bioavailable proteins.

For all the samples in this experimental setup, the moisture content increased during cultivation from an average initial value of 40 ± 0.9% to 65.9 ± 4.4% at the end of fermentation. This was likely due to loss of dry matter, generation of H_2_O during carbohydrate oxidation and the high humidity during cultivation [95]. The increase in moisture probably excludes some air necessary for the growth of the fungal mycelium. Hence, modifying the current SSF process used in this study to keep the moisture content around 40% has potential to result in an even higher protein increase in the final fungal product.

## 4. Conclusion

One innovative way to prevent stale bread and brewers spent grain (BSG) from going to waste is presented that convert these food by-products into nutritionally valuable products, enhancing their re-use in the food industry. Fungal SSF successfully bio transformed stale sourdough bread into a nutrient-enriched product. Most of the starch (65%) was converted into fungal protein (21%) with an improved amino acid composition. The fatty acid profile of the fermented product, reported here for the first time, revealed an increase in α-linolenic acid compared to the substrate. Moreover, SSF-induced increase in all minerals, vitamin E and ergocalciferol (vitamin D2); the latter which was almost absent prior to SSF. Addition of up to 12% brewers spent grain as substrate improved textural properties and the combined bread-BSG-fungi-products have great potential to meet the increasing demand from consumers looking for sustainable protein alternatives. Sensorial characteristics of the final products, scale-up trials, and validation of a cost-effective industrial process are proposed as a future step to take.

## References

[1] FAO. Global food losses and food waste – extent, causes and prevention. Rome: Food and Agriculture Organization of the United Nations; 2011.

[2] FAO. How to feed the world in 2050. Rome: Food and Agriculture Organization of the United Nations; 2009.

[3] Brancoli P, Rousta K, Bolton K. Life cycle assessment of supermarket food waste. ResouConserv Recycl. 2017;118:39–46.

[4] Hartmann C, Siegrist M. Consumer perception and behaviour regarding sustainable protein consumption: a systematic review. Trends Food SciTechnol. 2017;61:11–25.

[5] Steinfeld H, Gerber P, Wassenaar TD, et al. Livestock’s long shadow: environmental issues and options. Food and Agriculture Organization of the United Nations, USA; 2006.

[6] Godfray HCJ, Aveyard P, Garnett T, et al. Meat consumption, health, and the environment. Science. 2018;361(6399):eaam5324. 30026199 10.1126/science.aam5324

[7] Henchion M, Hayes M, Mullen A, et al. Future protein supply and demand: strategies and factors influencing a sustainable equilibrium. Foods. 2017;6(7):53. 10.3390/foods6070053 PMC5532560 28726744

[8] Wiebe M. Myco-protein from Fusarium venenatum: a well-established product for human consumption. Appl Microbiol Biotechnol. 2002;58(4):421–427. 11954786 10.1007/s00253-002-0931-x

[9] Knight N, Roberts G, Shelton D. The thermal stability of Quorn™ pieces. Int J Food Sci Technol. 2001;36(1):47–52.

[10] Bourne M. Food texture and viscosity: concept and measurement. Elsevier, US; 2002. p. 416.

[11] Takahashi T, Hayakawa F, Kumagai M, et al. Relations among mechanical properties, human bite parameters, and ease of chewing of solid foods with various textures. J Food Eng. 2009;95(3):400–409.

[12] Nout MJR, Kiers JL. Tempe fermentation, innovation and functionality: update into the third millenium. J Appl Microbiol. 2005;98(4):789–805. 15752324 10.1111/j.1365-2672.2004.02471.x

[13] Denardi-Souza T, Massarolo KC, Tralamazza SM, et al. Monitoring of fungal biomass changed by Rhizopus oryzae in relation to amino acid and essential fatty acids profile in soybean meal, wheat and rice. CyTA-J Food. 2018;16(1):156–164.

[14] Shurtleff W, Aoyagi A. The book of tempeh. Vol. 1. Soyinfo Center. New York, NY: Harper and Row; 1979.

[15] Blakeman JP, McCracken AR, Seaby DA. Changes brought about in solid substrates after fermentations of mixtures of cereals and pulses with Rhizopus oryzae. J Sci Food Agric. 1988;45(2):109–118.

[16] Hesseltine CW. Microbiology of oriental fermented foods. Annu Rev Microbiol. 1983;37(1):575–601. 6357061 10.1146/annurev.mi.37.100183.003043

[17] Vermeulen SJ, Wellesley L, Airey S, et al. Healthy diets from sustainable production: Indonesia. London: Chatham House; 2019.

[18] Souza Filho PF, Nair RB, Andersson D, et al. Vegan-mycoprotein concentrate from pea-processing industry byproduct using edible filamentous fungi. Fungal Biol Biotechnol. 2018;5(1):5. 29619233 10.1186/s40694-018-0050-9 PMC5880086

[19] Jin B, Yu Q, van Leeuwen JH, Hung YT. An Integrated Biotechnological Process for Fungal Biomass Protein Production and Wastewater Reclamation. In: Wang L, Tay JH, Tay S, Hung YT, editors. Environmental Bioengineering, vol 11. Totowa, NJ: Humana Press; 2010.

[20] Singh-Nee Nigam P, Singh D. Processing of agricultural wastes in solid state fermentation for microbial protein production. J Sci Ind Res. 1996;55(5–6):373–380.

[21] Barrios-González J. Solid-state fermentation: physiology of solid medium, its molecular basis and applications. Process Biochem. 2012;47(2):175–185.

[22] Hölker U, Höfer M, Lenz J. Biotechnological advantages of laboratory-scale solid-state fermentation with fungi. Appl Microbiol Biotechnol. 2004;64(2):175–186. 14963614 10.1007/s00253-003-1504-3

[23] Ferreira JA, Mahboubi A, Lennartsson PR, et al. Waste biorefineries using filamentous ascomycetes fungi: present status and future prospects. Bioresour Technol. 2016;215:334–345. 26996263 10.1016/j.biortech.2016.03.018

[24] Ferreira JA, Lennartsson PR, Edebo L, et al. Zygomycetes-based biorefinery: present status and future prospects. Bioresour Technol. 2013;135:523–532. 23127833 10.1016/j.biortech.2012.09.064

[25] Abu Yazid N, Barrena R, Komilis D, et al. Solid-state fermentation as a novel paradigm for organic waste valorization: a review. Sustainability. 2017;9(2):224.

[26] Melikoglu M, Webb C. Use of waste bread to produce fermentation products, in Food Industry Wastes, Kosseva MR, Webb C, Editors. San Diego: Academic Press; 2013. p. 63-76.

[27] Koutinas AA, Vlysidis A, Pleer D, et al. Valorization of industrial waste and by-product streams via fermentation for the production of chemicals and biopolymers. Chem Soc Rev. 2014;43(8):2587–2627. 24424298 10.1039/c3cs60293a

[28] Singhania RR, Patel AK, Soccol CR, et al. Recent advances in solid-state fermentation. Biochem Eng J. 2009;44(1):13–18.

[29] Krishna C. Solid-state fermentation systems—an overview. Crit Rev Biotechnol. 2005;25(1–2):1–30. 15999850 10.1080/07388550590925383

[30] Couto SR, Sanromán MA. Application of solid-state fermentation to food industry—a review. J Food Eng. 2006;76(3):291–302.

[31] Kitcha S, Cheirsilp B. Bioconversion of lignocellulosic palm byproducts into enzymes and lipid by newly isolated oleaginous fungi. Biochem Eng J. 2014;88:95–100.

[32] Kumar A, Roy B, Lakhani GP, et al. Evaluation of dried bread waste as feedstuff for growing crossbred pigs. group. 2014;2:0–377.

[33] Yuan B, Zhao L, Yang W, et al. Enrichment of bread with nutraceutical-rich mushrooms: impact of Auricularia auricula (Mushroom) flour upon quality attributes of wheat dough and bread. J Food Sci. 2017;82(9):2041–2050. 28753727 10.1111/1750-3841.13812

[34] Brancoli P, Lundin M, Bolton K, et al. Bread loss rates at the supplier-retailer interface–analysis of risk factors to support waste prevention measures. ResouConserv Recycl. 2019;147:128–136.

[35] Melikoglu M. Production of sustainable alternatives to petrochemicals and fuels using waste bread as a raw material. School of Chemical Engineering and Analytical Science; 2008, University of Manchester. p. 320.

[36] Ebrahimi F, Khanahmadi M, Roodpeyma S, et al. Ethanol production from bread residues. Biomass Bioenergy. 2008;32(4):333–337.

[37] Melikoglu M, Lin CSK, Webb C. Solid state fermentation of waste bread pieces by Aspergillus awamori: analysing the effects of airflow rate on enzyme production in packed bed bioreactors. Food Bioprod Process. 2015;95:63–75.

[38] Kiran EU, Trzcinski AP, Liu Y. Glucoamylase production from food waste by solid state fermentation and its evaluation in the hydrolysis of domestic food waste. Biofuel Res J. 2014;1(3):98–105.

[39] Cerda A, El-Bakry M, Gea T, et al. Long term enhanced solid-state fermentation: inoculation strategies for amylase production from soy and bread wastes by Thermomyces sp. in a sequential batch operation. J Environ Chem Eng. 2016;4(2):2394–2401.

[40] Wang R, Godoy LC, Shaarani SM, et al. Improving wheat flour hydrolysis by an enzyme mixture from solid state fungal fermentation. Enzyme Microb Technol. 2009;44(4):223–228.

[41] Mamma D, Kourtoglou E, Christakopoulos P. Fungal multienzyme production on industrial by-products of the citrus-processing industry. Bioresour Technol. 2008;99(7):2373–2383. 17604624 10.1016/j.biortech.2007.05.018

[42] Verni M, Rizzello CG, Coda R. Fermentation biotechnology applied to cereal industry by-products: nutritional and functional insights. Front Nutr. 2019;6. DOI:10.3389/fnut.2019.00042 PMC6473998 31032259

[43] Lynch KM, Steffen EJ, Arendt EK. Brewers’ spent grain: a review with an emphasis on food and health. J Inst Brewing. 2016;122(4):553–568.

[44] Xiros C, Christakopoulos P. Biotechnological potential of brewers spent grain and its recent applications. Waste Biomass Valorization. 2012;3(2):213–232.

[45] Canedo MS, de Paula FG, da Silva FA, et al. Protein enrichment of brewery spent grain from Rhizopus oligosporus by solid-state fermentation. Bioprocess Biosyst Eng. 2016;39(7):1105–1113. 26984742 10.1007/s00449-016-1587-8

[46] Gmoser R, Sintca C, Taherzadeh MJ, et al. Combining submerged and solid state fermentation to convert waste bread into protein and pigment using the edible filamentous fungus N. intermedia. Waste Manage. 2019;97:63–70. 10.1016/j.wasman.2019.07.039 31447028

[47] Handoyo T, Morita N. Structural and functional properties of fermented soybean (tempeh) by using Rhizopus oligosporus. Int J Food Prop. 2006;9(2):347–355.

[48] The Swedish Food Composition Database. The Swedish food composition database. 2017.

[49] Sluiter A, Hames B, Ruiz R, et al. Determination of structural carbohydrates and lignin in biomass. Lab Anal Proced. 2008;1617:1–16.

[50] Hinchcliffe J, Carlsson NG, Jönsson E, et al. Aquafeed ingredient production from herring (Clupea harengus) by-products using pH-shift processing: effect from by-product combinations, protein solubilization-pH and centrifugation force. Anim Feed Sci Technol. 2019;247:273–284.

[51] Barbarino E, Oiano-Neto J, et al. Gross chemical profile and calculation of nitrogen-to-protein conversion factors for five tropical seaweeds. Am J Plant Sci. 2011;2(3):287-296.

[52] Lee CM, Trevino B, Chaiyawat M. A simple and rapid solvent extraction method for determining total lipids in fish tissue. J AOAC Int. 1996;79(2):487–492. 8920137

[53] Lykkesfeldt J. Determination of ascorbic acid and dehydroascorbic acid in biological samples by high-performance liquid chromatography using subtraction methods: reliable reduction with tris [2-carboxyethyl] phosphine hydrochloride. Anal Biochem. 2000;282(1):89–93. 10860503 10.1006/abio.2000.4592

[54] Larsson K, Almgren A, Undeland I. Hemoglobin-mediated lipid oxidation and compositional characteristics of washed fish mince model systems made from cod (Gadus morhua), herring (Clupea harengus), and salmon (Salmo salar) muscle. J Agric Food Chem. 2007;55(22):9027–9035. 17910510 10.1021/jf070522z

[55] Standal IB, Mozuraityte R, Rustad T, et al. Quality of filleted Atlantic Mackerel (Scomber Scombrus) during chilled and frozen storage: changes in lipids, Vitamin D, proteins, and small metabolites, including Biogenic Amines. J Aquatic Food Prod Technol. 2018;27(3):338–357.

[56] Jäpelt RB, Silvestro D, Smedsgaard J, et al. LC–MS/MS with atmospheric pressure chemical ionisation to study the effect of UV treatment on the formation of vitamin D3 and sterols in plants. Food Chem. 2011;129(1):217–225.

[57] Kim K, Choi B, Lee I, et al. Bioproduction of mushroom mycelium of Agaricus bisporus by commercial submerged fermentation for the production of meat analogue. J Sci Food Agric. 2011;91(9):1561–1568. 21445867 10.1002/jsfa.4348

[58] Guinard J-X, Mazzucchelli R. The sensory perception of texture and mouthfeel. Trends Food SciTechnol. 1996;7(7):213–219.

[59] Asgar MA, Fazilah A, Huda N, et al. Nonmeat protein alternatives as meat extenders and meat analogs. Compr Rev Food Sci Food Saf. 2010;9(5):513–529. 33467834 10.1111/j.1541-4337.2010.00124.x

[60] WHO/FAO/UNU. Protein and amino acid requirements in human nutrition. Report of a Joint WHO/FAO/UNU Expert Consultation, 2007, p. 935. 18330140

[61] Waters DM, Jacob F, Titze J, et al. Fibre, protein and mineral fortification of wheat bread through milled and fermented brewer’s spent grain enrichment. European Food Res and Tech. 2012;235(5):767–778.

[62] Shojaosadati SA, Faraidouni R, Madadi-Nouei A, et al. Protein enrichment of lignocellulosic substrates by solid state fermentation using Neurospora sitophila. ResouConserv Recycl. 1999;27(1–2):73–87.

[63] Starzyńska-Janiszewska A, Stodolak B, Duliński R, et al. Fermentation of colored Quinoa seeds with Neurospora intermedia to obtain oncom-type products of favorable nutritional and bioactive characteristics. Cereal Chemistry Journal. 2017;94(3):619–624.

[64] Wang D, Sakoda A, Suzuki M. Biological efficiency and nutritional value of Pleurotus ostreatus cultivated on spent beer grain. Bioresour Technol. 2001;78(3):293–300. 11341691 10.1016/s0960-8524(01)00002-5

[65] Karimi S, Mahboobi Soofiani N, Lundh T, et al. Evaluation of filamentous fungal biomass cultivated on Vinasse as an alternative nutrient source of fish feed: protein, lipid, and mineral composition. Fermentation. 2019;5(4):99.

[66] Nuraini S, Latif SA. Improving the quality of tapioca by product through fermentation by neurospora crassa to produce $ carotene rich feed. Pak J Nutr. 2009;8(4):487–490.

[67] Nuraini AD, Mahata ME. Improving the nutrient quality of durian (Durio zibethinus) fruit waste through fermentation by using Phanerochaete chrysosporium and Neurospora crassa for poultry diet. Int J Poult Sci. 2015;14:354–358.

[68] Trinci APJ. Evolution of the Quorn® myco-protein fungus, Fusarium graminearum A3/5. Microbiology. 1994;140(9):2181–2188. 7952168 10.1099/13500872-140-9-2181

[69] Liu P, Li J, Deng Z. Bio-transformation of agri-food wastes by newly isolated Neurospora crassa and Lactobacillus plantarum for egg production. Poult Sci. 2016;95(3):684–693. 26740129 10.3382/ps/pev357

[70] Chan LG, Cohen JL, de Moura Bell JMLN. Conversion of agricultural streams and food-processing by-products to value-added compounds using filamentous fungi. Annu Rev Food Sci Technol. 2018;9:503–523. 29328807 10.1146/annurev-food-030117-012626

[71] Ferreira JA, Lennartsson PR, Taherzadeh MJ. Production of ethanol and biomass from thin stillage by Neurospora intermedia: a pilot study for process diversification. Eng Life Sci. 2015;15(8):751–759.

[72] Abu OA, Tewe OO, Losel DM, et al. Changes in lipid, fatty acids and protein composition of sweet potato (Ipomoea batatas) after solid-state fungal fermentation. Bioresour Technol. 2000;72(2):189–192.

[73] Matsuo M. Preparation and components of okara-ontjom, a traditional Indonesian fermented food. Nippon Shokuhin Kogyo Gakkai-Shi. 1997;44(9):632–639.

[74] Oduguwa OO, Edema MO, Ayeni AO. Physico-chemical and microbiological analyses of fermented corn cob, rice bran and cowpea husk for use in composite rabbit feed. Bioresour Technol. 2008;99(6):1816–1820. 17502134 10.1016/j.biortech.2007.03.036

[75] Dos Santos Oliveira M, Feddern V, Kupski L, et al. Changes in lipid, fatty acids and phospholipids composition of whole rice bran after solid-state fungal fermentation. Bioresour Technol. 2011;102(17):8335–8338. 21715163 10.1016/j.biortech.2011.06.025

[76] Graham DC, Steinkraus KH, Hackler LR. Factors affecting production of mold mycelium and protein in synthetic media. Appl Environ Microbiol. 1976;32(3):381–387. 10836 10.1128/aem.32.3.381-387.1976 PMC170074

[77] Beuchat LR, Worthington RE. Changes in the lipid content of fermented peanuts. J Agric Food Chem. 1974;22(3):509–512.

[78] McKeon TA, Goodrich-Tanrikulu M, Lin J-T, et al. Pathways for fatty acid elongation and desaturation in Neurospora crassa. Lipids. 1997;32(1):1–5. 9075186 10.1007/s11745-997-0001-8

[79] Zhao G, Etherton TD, Martin KR, et al. Dietary α-linolenic acid reduces inflammatory and lipid cardiovascular risk factors in hypercholesterolemic men and women. J Nutr. 2004;134(11):2991–2997. 15514264 10.1093/jn/134.11.2991

[80] Čertík M, Adamechová Z, Guothová L. Simultaneous enrichment of cereals with polyunsaturated fatty acids and pigments by fungal solid state fermentations. J Biotechnol. 2013;168(2):130–134. 23583333 10.1016/j.jbiotec.2013.03.016

[81] Oliveira MDS, Feddern V, Kupski L, et al. Physico-chemical characterization of fermented rice bran biomass Caracterización fisico-química de la biomasa del salvado de arroz fermentado. CyTA–J Food. 2010;8(3):229–236.

[82] Becker W, Lyhne N, Pedersen AN, et al. Nordic nutrition recommendations 2004-integrating nutrition and physical activity. Scand J Nutr. 2004;48(4):178–187.

[83] European Food Safety. Dietary reference values for nutrients summary report. EFSA Supporting Publications. 2017;14(12):e15121E.

[84] Combs GF Jr, McClung JP. The vitamins: fundamental aspects in nutrition and health. US: Academic press; 2016.

[85] Howarth NC, Saltzman E, Roberts SB. Dietary fiber and weight regulation. Nutr Rev. 2001;59(5):129–139. 11396693 10.1111/j.1753-4887.2001.tb07001.x

[86] National Food Agency, S. Search for nutrients. 2017 [cited 2019 Jun 10]. Available from: http://www7.slv.se/SokNaringsinnehall/

[87] Garcıa ML, Dominguez R, Galvez MD, et al. Utilization of cereal and fruit fibres in low fat dry fermented sausages. Meat Sci. 2002;60(3):227–236. 22063393 10.1016/s0309-1740(01)00125-5

[88] Quorn. Sustainable future of food – production of first-class protein alternative for a balanced diet. Marlow Foods Ltd, UK; 2017.

[89] Moo-Young M, Chisti Y, Vlach D. Fermentation of cellulosic materials to mycoprotein foods. Biotechnol Adv. 1993;11(3):469–479. 14545669 10.1016/0734-9750(93)90015-f

[90] Pastore GM, Park YK, Min DB. Production of fruity aroma by Neurospora from beiju. Mycol Res. 1994;98(11):1300–1302.

[91] Walker GM, White NA. Introduction to fungal physiology. Fungi: biology and applications; 2017. p. 1–35.

[92] Wang HL, Swain EW, Hesseltine CW. Mass production of Rhizopus oligosporus spores and their applicaton in tempeh fermentation. 1975.

[93] Melikoglu M, Lin CSK, Webb C. Stepwise optimisation of enzyme production in solid state fermentation of waste bread pieces. Food Bioprod Process. 2013;91(4):638–646.

[94] Cantabrana I, Perise R, Hernández I. Uses of Rhizopus oryzae in the kitchen. Int J Gastronomy Food Sci. 2015;2(2):103–111.

[95] Raimbault M, Alazard D. Culture method to study fungal growth in solid fermentation. Eur J Appl Microbiol Biotechnol. 1980;9(3):199–209.

